# Supplementary figures and images for: Support needs and barriers to accessing support: Baseline results of a mixed-methods national survey of people bereaved during the COVID-19 pandemic
Source: Palliat Med. 2021 Oct 22;35(10):1985–97. doi: 10.1177/02692163211043372 (PMC8637353; doi:10.1177/02692163211043372)

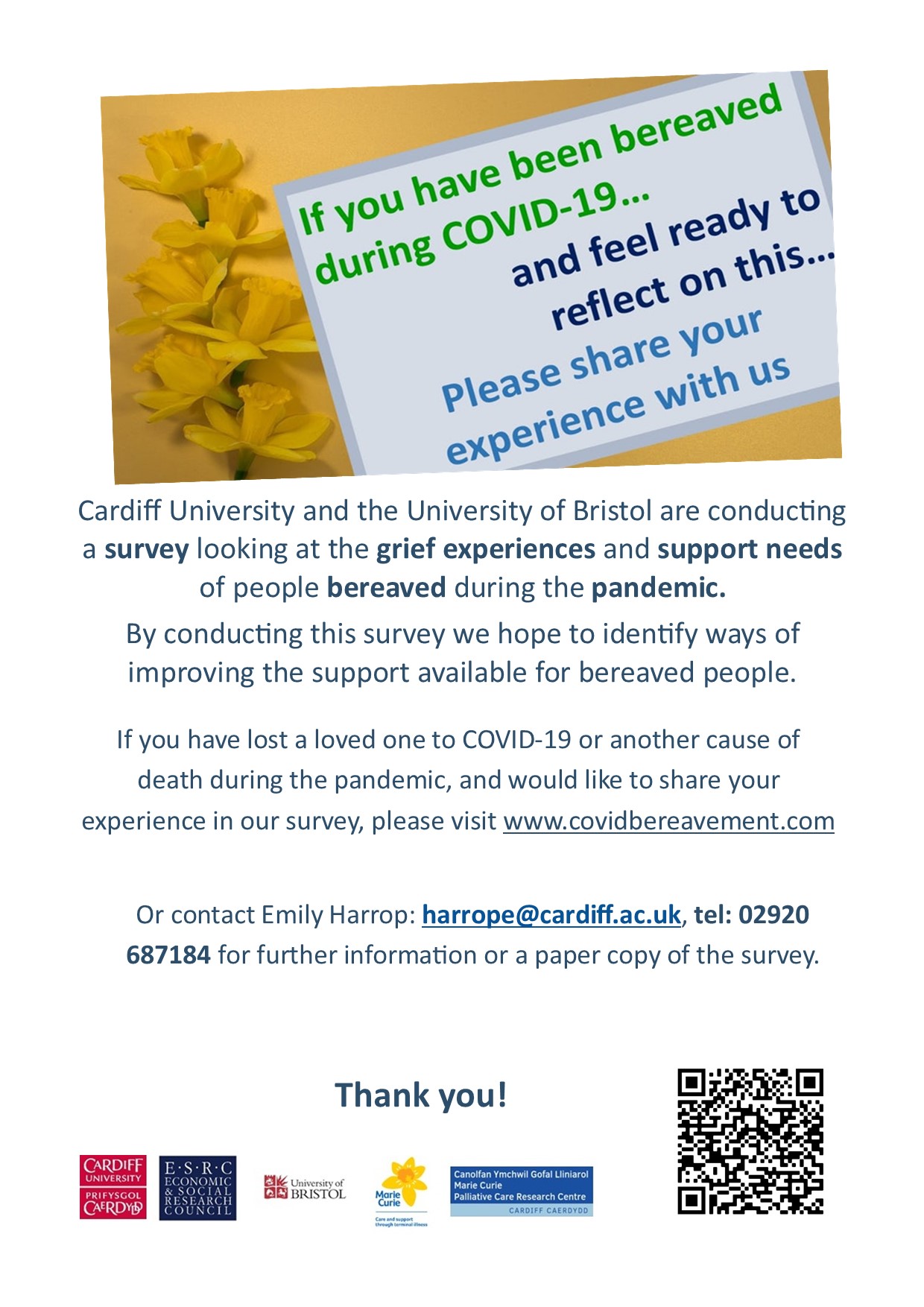

Supplement: sj-jpg-2-pmj-10.1177_02692163211043372 – Supplemental material for Support needs and barriers to accessing support: Baseline results of a mixed-methods national survey of people bereaved during the COVID-19 pandemic [file sj-jpg-2-pmj-10.1177_02692163211043372.jpg]
